# A novel puromycin decorporation method to quantify skeletal muscle protein breakdown: A proof-of-concept study

**DOI:** 10.1016/j.bbrc.2017.10.085

**Published:** 2017-12-16

**Authors:** Hannah Crossland, Kenneth Smith, Philip J. Atherton, Daniel J. Wilkinson

**Affiliations:** MRC-ARUK Centre for Musculoskeletal Ageing Research, National Institute for Health Research (NIHR) Biomedical Research Centre (BRC), Clinical, Metabolic and Molecular Physiology, University of Nottingham, Royal Derby Hospital, Derby, UK

**Keywords:** Skeletal muscle, Protein breakdown, Puromycin

## Abstract

The precise roles that the major proteolytic pathways play in the regulation of skeletal muscle mass remain incompletely understood, in part due to technical limitations associated with current techniques used to quantify muscle protein breakdown (MPB). We aimed to develop a method to assess MPB in cells, based on loss of puromycin labelling of translated polypeptide chains. Following an initial 24 h incubation period with puromycin (1 μM), loss of puromycin labelling from murine C2C12 myotubes was assessed over 48 h, both in the presence or absence of protein synthesis inhibitor cycloheximide (CHX). To validate the method, loss of puromycin labelling was determined from cells treated with selected compounds known to influence MPB (e.g. serum starvation, Dexamethasone (Dex), tumour necrosis factor alpha (TNF-α) and MG-132)). Reported established (static) markers of MPB were measured following each treatment. Loss of puromycin labelling from cells pre-incubated with puromycin was evident over a 48 h period, both with and without CHX. Treatment with Dex (−14 ± 2% vs. Ctl; *P* < 0.01), TNF-α (−20 ± 4% vs. Ctl; *P* < 0.001) and serum starvation (−14 ± 4% vs. Ctl; *P* < 0.01) caused a greater loss of puromycin labelling than untreated controls, while the proteasome inhibitor MG-132 caused a relatively lower loss of puromycin labelling (+15 ± 8% vs. Ctl; *P* < 0.05). Thus, we have developed a novel decorporation method for measuring global changes in MPB, validated *in vitro* using an established muscle cell line. It is anticipated this non isotopic-tracer alternative to measuring MPB will facilitate insight into the mechanisms that regulate muscle mass/MPB both *in vitro*, and perhaps, *in vivo*.

## Highlights

• Limitations exist in the techniques used to quantify muscle protein breakdown (MPB).
• We developed a method for assessing MPB through loss of puromycin labelling in cells.
• We validated the method using selected compounds known to dynamically modulate MPB.

## 1 Introduction

Several proteolytic pathways exist in skeletal muscle to regulate protein breakdown and maintain protein homeostasis. The ubiquitin-proteasome pathway (UPP) plays a major role in regulating muscle protein breakdown (MPB), where proteins targeted for degradation by the UPP are initially polyubiquitinated by covalent attachment of a chain of ubiquitin molecules, which are then recognised by the 26S proteasome for degradation [1], [2]. Caspases and calcium-activated calpains also contribute to protein breakdown in muscle, where it has been proposed that they may cleave myofibrillar proteins in order to make them more accessible to degradation by the UPP [3], [4], [5], [6]. The autophagy-lysosome system also represents a major mechanism for protein degradation, responsible for breakdown of organelles as well as bulk cytoplasmic and long-lived proteins [1], [7].

While activation of the above proteolytic pathways has been reported in muscle during various catabolic conditions, including sepsis, fasting, sarcopenia and cachexia [2], [3], [6], [7], methodological difficulties have limited (versus muscle protein synthesis (MPS)) understanding of the role that MPB plays in these conditions. That being said a number of techniques exist. For instance, 3-methylhistidine (3-MH) [8] is a post-translational methylation of histidine resulting from actin and myosin degradation. Since 3-MH cannot be further metabolized or reincorporated, it has been used as a marker of myofibrillar proteolysis, and thus MPB [8], [9], [10]. However, limitations exist with this method, since 3-MH can also be produced by other tissue types as well as from dietary meat protein metabolism, while low concentrations make the quantification of 3-MH challenging [11], [12]. Measurement of the rate of release of labelled amino acid(s) from tissue/cells is another method that has been used to assess MPB. Infusion or incubation with a radiolabelled or stable isotope of an amino acid that is neither made nor catabolized by muscle (e.g. tyrosine), and its subsequent release from tissue/cells under conditions where reincorporation is inhibited, can be used to estimate MPB [13]; often requiring specialist equipment. Moreover, issues can arise when labelled amino acids released during proteolysis are recycled for MPS, giving rise to an underestimation of MPB rates, and these methods require specialized equipment for measurement.

A number of years ago, the surface sensing of translation (SUnSET) technique [14], [15] was developed to study cellular global protein synthesis both in cultured cells *in vitro* and *in vivo* animal models as an alternative to classical radiolabelling or stable isotope methods. Rather than using tracers to measure incorporation of a labelled amino acid into protein, this technique involves incubation with puromycin, an antibiotic that is a structural analogue of tyrosyl-tRNA, which can be incorporated into a translating polypeptide chain [14]. Since incorporation of puromycin into a nascent peptide chains results in the termination of peptide elongation, the rate at which puromycin-labelled polypeptides are formed (anti-puromycin antibodies serve to detect puromycin-conjugated peptides) acts as an effective measure of global rates of protein synthesis.

Crucially, as previously alluded to, a key advantage of the SUnSET method is that it does not require expensive/specialized equipment and techniques; instead only common laboratory methodologies, i.e. Western blot or immunohistochemistry. A method for assessing MPB using similar non-tracer techniques would undoubtedly be advantageous for research into muscle protein metabolism. In this study, we hypothesised that a puromyocin decorporation approach could be used for measuring global changes in MPB i.e. following incubation periods with puromycin, a subsequent decrease in puromycin labelling in cells would be indicative of a change in MPB. We developed this method *in vitro* using murine C2C12 myotubes, and validated the approach using selected compounds known to dynamically modulate MPB.

## 2 Materials and methods

### 2.1 C2C12 cell culture

Mouse C2C12 myoblasts (ECACC, Salisbury, UK) were cultured in Dulbecco's Modified Eagle Medium (DMEM; Thermo Fisher Scientific) supplemented with 10% (v/v) fetal bovine serum (FBS), 1% (v/v) antibiotic-antimycotic solution and 4 mM l-glutamine (all from Sigma-Aldrich, UK) and maintained at 37 °C with 5% CO_2_. For experimentation, myoblasts (between passage 6 and 8) were seeded onto six-well dishes (Nunclon™ Delta; Thermo Fisher Scientific) and at ∼90% confluency, differentiation was initiated by changing the medium to DMEM supplemented with 2% (v/v) horse serum, 1% (v/v) antibiotic-antimycotic solution and 4 mM l-glutamine (Sigma-Aldrich, UK). The media was replaced every 48 h.

### 2.2 Puromycin MPB method

Experiments were performed on day 4–5 post induction of differentiation. Initial experiments assessed the time course of puromycin incorporation into cellular protein, and its disappearance from cells upon removal from the culture media, both in the presence and absence of cycloheximide (CHX; final concentration 25 ng/ml). For puromycin incorporation, cells were incubated for 1–24 h with puromycin (final concentration 1  μM), after which cells were harvested in homogenisation buffer for Western blot analysis (see below). To determine the rate of puromycin loss from cells, myotubes were incubated for 24 h with puromycin, then cells were washed twice with Hanks' Balanced Salt Solution (HBSS), and replaced with media lacking puromycin (with or without CHX). Cells were harvested at various times points up until 48 h post removal of puromycin.

For validation of the method, cells were incubated with selected treatments known to influence muscle protein breakdown. Myotubes were incubated for 24 h with puromycin, after which cells were washed with HBSS and fresh media (without puromycin, and with or without CHX) replaced. At this point, cells were subjected to the following treatments: serum starvation, dexamethasone (Dex; 1 μM; Sigma-Aldrich, UK), tumour necrosis factor alpha (TNF-α; 10 ng/ml; Sigma-Aldrich, UK) or MG-132 (20 μM; Sigma-Aldrich, UK). Samples were harvested after 4 h or 24 h treatment. All cells were harvested by removing the media, washing twice with ice-cold PBS, and collecting in homogenisation buffer (50 mM Tris-HCl, pH7.5, 1 mM EDTA, 1 mM EGTA, 10 mM β-glycerophosphate, 50 mM NaF and complete protease inhibitor cocktail tablet (Roche, UK). n = 4–6 technical well replicates were used for each treatment group.

### 2.3 Western blotting

Cell lysates were prepared by passing samples repeatedly through gel-loading pipette tips. Samples were centrifuged (13,000 g for 10 min, 4 °C) and lysates (10 μg total protein) were electrophoresed using Criterion XT 12% Bis-Tris gels (Bio-Rad, UK) at 200 V for 1 h. Samples were transferred to PVDF membranes at 100 V for 45 min, and membranes were blocked in 5% (w/v) milk for 1 h at room temperature. Membranes were incubated overnight at 4 °C in the presence of the following primary antibodies: puromycin (1:20,000; Millipore), ubiquitin (1:2000; Cell Signaling Technology), MAFbx (1:500), MuRF1 (1:2000; ECM Biosciences), Calpain 1 (1:2000; Abcam), ULK1 Ser555 (1:2000; Cell Signaling Technology) and Raptor Ser792 (1:2000; Cell Signaling Technology). The following day, membranes were washed for 3 × 5 min with TBS-Tween then incubated in HRP-conjugated secondary antibody (anti-mouse 1:10,000 for puromycin; anti-mouse 1:2000 for Calpain 1; anti-rabbit 1:2000 for ubiquitin, MAFbx and MuRF1) for 1 h at room temperature. Membranes were again washed in TBS-Tween and bands were detected using Chemiluminescent HRP Substrate (Millipore EMD) on a Chemidoc XRS imaging system (Bio-Rad, UK). Bands were normalized against total protein loading by Coomassie staining the membrane.

### 2.4 Statistical analyses

All data are presented as means ± SEM and analyzed using GraphPad Prism version 6 (La Jolla, USA). Unpaired *t* tests were used to compare differences between treatments and untreated controls. For the time course data, two-way ANOVA with Tukey's multiple comparison test was used to assess differences between time points and CHX treatment. *P* < 0.05 was taken to be statistically significant.

## 3 Results

### 3.1 Time course of puromycin incorporation into, and disappearance from, C2C12 cells

**Fig. 1:**
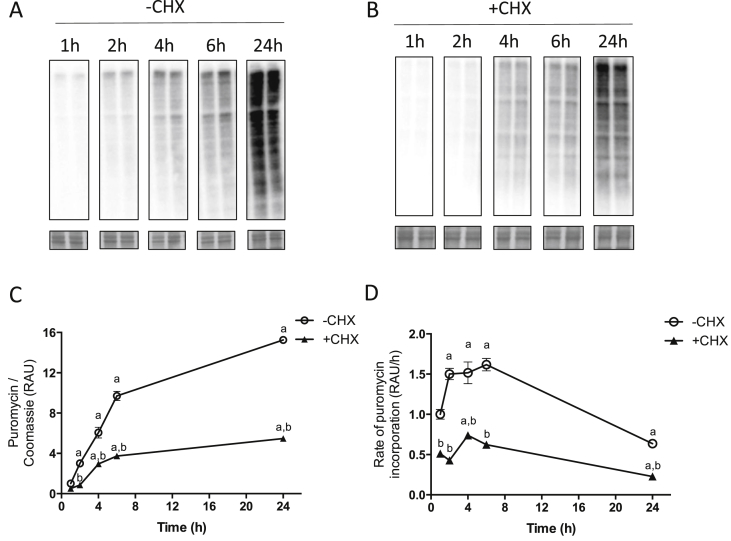
**Time course of puromycin incorporation into C2C12 myotubes.** C2C12 myotubes were incubated for various times with puromycin, without (A) or with (B) the addition of cycloheximide (CHX). A & B, representative blots for puromycin labelling over time. Coomassie stained membranes are shown below. C, Puromycin labelling in cells over time, expressed as relative arbitrary units (RAU), normalized to Coomassie and relative to ‘1 h –CHX’. D, Rate of puromycin incorporation into cells, expressed as RAU per hour. Data are expressed as mean ± SEM, n = 4 well replicates for each time point. a; *P* < 0.05 vs. 1 h time point. B; *P* < 0.05 vs. –CHX.

C2C12 cells were harvested at various time points after addition of puromycin to determine incorporation over a 24 h period. In the absence of CHX, puromycin incorporation increased significantly over 24 h (+1425 ± 42%; *P* < 0.001 vs. 1 h at 24 h; Fig. 1A, C), with a linear rate of incorporation during 2 h-6h (Fig. 1D). With CHX, puromycin incorporation was significantly lower than in cells without CHX at all time points, although there was a significant increase in labelling by 4 h (+196 ± 39%; *P* < 0.001 vs. 1 h; Fig. 1B and C) and by 24 h was a 466 ± 23% increase in puromycin incorporation (*P* < 0.001 vs. 1 h).

**Fig. 2:**
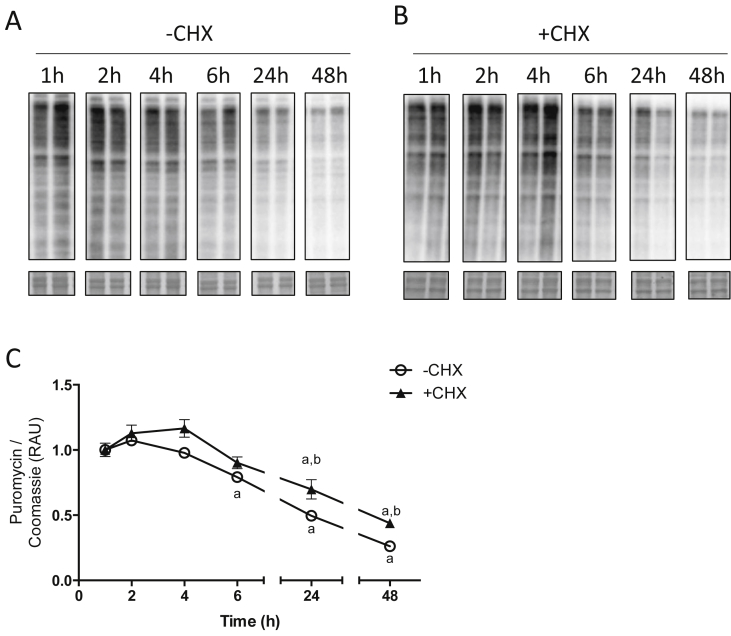
**Time course of puromycin disappearance in C2C12 cells after removal from the culture media.** Cells were incubated for 24 h with puromycin, after which puromycin was removed from the media and cells were harvested at various time points, without (A) or with (B) cycloheximide (CHX), for analysis of puromycin labelling. Coomassie stained membranes are shown below. C, Puromycin labelling in cells, expressed as relative arbitrary units (RAU), normalized to Coomassie and relative to corresponding 1 h time point. Data are expressed as mean ± SEM, n = 4 well replicates for each time point. a; *P* < 0.05 vs. 1 h time point. B; *P* < 0.05 vs. –CHX.

24 h was selected as the initial incubation period for assessing MPB. Cells were treated for 24 h with puromycin, washed, and fresh media replaced to assess loss of puromycin labelling over time. During the initial 4 h after puromycin removal, no loss of labelling was observed (Fig. 2A, C), but from 6 h until 48 h there was a gradual decrease in puromycin-labelled polypeptides within the cells (−74 ± 2%; at 48 h; P < 0.001 vs. 1 h; Fig. 2A, C). Inclusion of CHX at the point of puromycin removal did not prevent the loss of puromycin from cells, although there was a delayed decline in labelling, with significant decreases in puromycin labelling from 24 h onwards (−66 ± 3%; at 48 h; *P* < 0.001 vs. 1 h; Fig. 2B and C).

### 3.2 Effects of selected treatments on loss of puromycin labelling in C2C12 cells

**Fig. 3:**
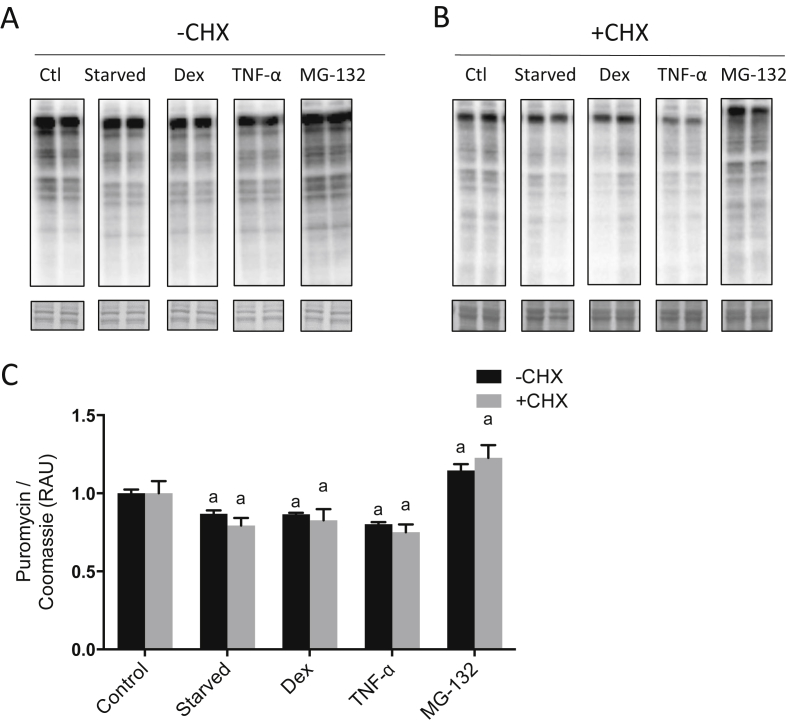
**Puromycin disappearance in C2C12 cells following treatments known to influence protein breakdown.** Cells were incubated for 24 h with puromycin, after which puromycin was removed from the media and cells were treated: with serum starvation, dexamethasone (Dex; 1 μM), tumour necrosis factor-α (TNF-α; 10 ng/ml) or MG-132 (20 μM) for 24 h. Experiments were performed in the absence (A) or presence (B) of cycloheximide (CHX), for analysis of puromycin labelling. Coomassie stained membranes are shown below. C, Puromycin labelling in cells, expressed as relative arbitrary units (RAU), normalized to Coomassie and relative to untreated controls. Data are expressed as mean ± SEM, n = 6 well replicates for each group. a; *P* < 0.05 vs. control.

Selected treatments known to influence muscle protein breakdown (serum starvation, Dex, TNF-α and MG-132) were used to validate the method. Puromycin was removed from the media after 24 h incorporation, at which point each treatment was added. Following 24 h serum starvation, Dex and TNF-α, there was a greater loss of cellular puromycin than in untreated cells (−14 ± 4%; *P* < 0.01 vs. Ctl; −14 ± 2%; *P* < 0.01 vs. Ctl; −20 ± 4%; *P* < 0.001 vs. Ctl, respectively, Fig. 3A, C). With MG-132 treatment, there was a relatively lower loss of puromycin labelling compared to untreated controls (+15 ± 8%; *P* < 0.05 vs. Ctl; Fig. 3A, C). A similar pattern of changes was observed with each treatment when CHX was included at the onset of treatment (Fig. 3B and C).

### 3.3 Markers of MPB following incubation with treatments known to influence MPB

**Fig. 4:**
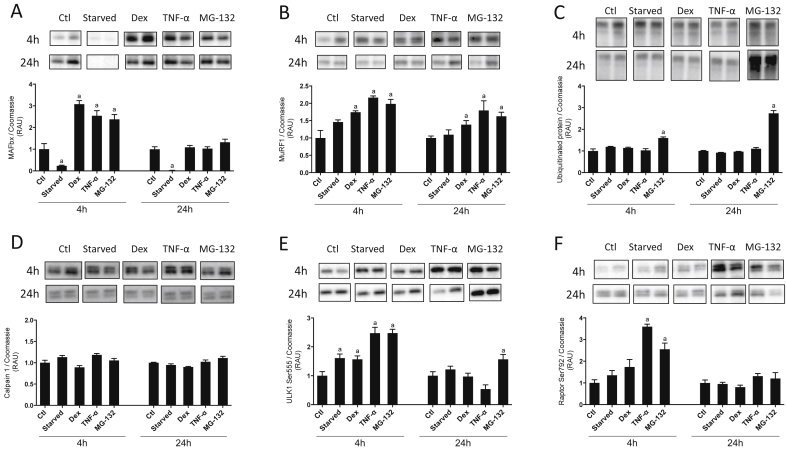
**Markers of muscle protein breakdown (MPB) in C2C12 cells following incubation with various treatments known to influence MPB.** Cells were incubated for 24 h with puromycin, after which puromycin was removed from the media and cells were treated with: serum starvation, dexamethasone (Dex; 1 μM), tumour necrosis factor-α (TNF-α; 10 ng/ml) or MG-132 (20 μM) for 4 h or 24 h. Lysates were analyzed for total MAFbx (A), MuRF1 (B), ubiquitinated protein (C), Calpain 1 (D), ULK1 Ser555 (E) and Raptor Ser792 (F) expression. Data are expressed as mean ± SEM relative to untreated controls and normalized to Coomassie, n = 4–5 well replicates for each group. Representative blots are shown above each graph. a; *P* < 0.05 vs. control.

MAFbx protein expression was increased following 4 h Dex (+207 ± 34%; *P* < 0.001 vs. Ctl), TNF-α (+154 ± 47%; *P* < 0.01 vs. Ctl) and MG-132 (+138 ± 44%; *P* < 0.01 vs. Ctl) treatment (Fig. 4A), while serum starvation caused a reduction in MAFbx protein (−76 ± 5%; *P* < 0.001 vs. Ctl). After 24 h, MAFbx protein was no different from controls for all treatments except for serum starvation, where it was decreased relative to control (−97 ± 0%; *P* < 0.001 vs. Ctl; Fig. 4A). Protein expression of MuRF1 was unaffected by serum starvation (Fig. 4B) but was increased at 4 h and 24 h with Dex (+74 ± 8%; *P* < 0.05 vs. Ctl at 4 h, +38 ± 24%; *P* < 0.01 vs. Ctl at 24 h), TNF-α (+117 ± 9%; *P* < 0.01 vs. Ctl at 4 h, +80 ± 55%; *P* < 0.05 vs. Ctl at 24 h) and MG-132 (+98 ± 26%; *P* < 0.01 vs. Ctl at 4 h, +63 ± 23%; *P* < 0.01 vs. Ctl at 24 h). Total ubiquitinated protein was unaffected by each treatment except for with MG-132, where there was a significant increase at both time points (+60 ± 10%; *P* < 0.01 vs. Ctl at 4 h, +174 ± 25%; *P* < 0.001 vs. Ctl at 24 h; Fig. 4C).

Calpain 1 protein was not significantly altered at either time point with any of the treatments (Fig. 4D). Phosphorylation of ULK1 (Ser555; Fig. 4E) was significantly increased at 4 h by serum starvation (+61 ± 11%; *P* < 0.05 vs. Ctl), Dex (+57 ± 10%; *P* < 0.05 vs. Ctl), TNF-α (+147 ± 17%; *P* < 0.001 vs. Ctl) and MG-132 (+148 ± 11%; *P* < 0.001 vs. Ctl). At 24 h, ULK1 Ser555 was increased relative to controls with MG-132 (+57 ± 14%; *P* < 0.05 vs. Ctl). Raptor phosphorylation (Ser792; Fig. 4F) was increased following 4 h TNF-α (+260 ± 9%; *P* < 0.001 vs. Ctl) and MG-132 (+155 ± 23%; *P* < 0.001 vs. Ctl) treatments, while after 24 h, Raptor phosphorylation was no different from control for all treatments (Fig. 4F).

## 4 Discussion

Although much insight has been acquired into the molecules and mechanisms involved in maintaining protein homeostasis in skeletal muscle during health and during catabolic conditions such as sarcopenia, cachexia, and disuse, ubiquitous methodological difficulties in measuring MPB have somewhat limited progress. The SunSET technique [14], based on the measurement of the rate of formation of puromycin labelled peptides, has been established as a valid and accurate approach for assessing protein synthesis in multiple tissue types [16]. In the present study, we aimed to determine whether following an initial incubation, loss of puromycin labelling from cells could be used to measure changes in protein breakdown. We developed this method *in vitro* using C2C12 myotubes, with selected treatments known to influence MPB to validate the method.

During initial testing of our hypothesis, we determined the optimal initial incubation period for puromycin incorporation in our chosen model. In doing so, we observed that during the first 6 h puromycin incorporation was linear, and by 24 h, incorporation rates appeared to be declining i.e. potentially reaching a level of saturation. For this reason, we chose an initial incubation period of 24 h. Subsequent loss of cellular puromycin-conjugated polypeptides was monitored over a 48 h time course. During the initial 4–6 h period after puromycin removal, no loss of labelling was observed. This could feasibly be due to a lack of breakdown during this period, potentially through the addition of fresh media to cells inhibiting protein breakdown (i.e. ‘feed’), but may also have been due to ‘saturation’ of the puromycin signal. It is also feasible that during this period there was still some incorporation of remaining intracellular puromycin. From 6 to 48 h however, there was a continual decline in puromycin labelling.

From these findings, 24 h was selected for validating this method, but we suggest that for future studies, the end user should optimise conditions to suit cell type and individual experimental conditions. We also assessed loss of puromycin labelling in C2C12 cells in the presence of CHX (added at the point of puromycin removal) to eliminate the possibility that the observed changes were influenced by changes in MPS (i.e. reincorporation). It should be noted that the dose of CHX that we used did not completely inhibit MPS (Fig. 1); however higher doses caused cell viability issues after 24 h (cells in suspension), which would clearly compromise the true experimental readouts of MPB. Moreover, the addition of CHX did not prevent the decline in puromycin labelling across 48 h (although by 48 h there was a smaller decrease in labelling compared to cells without CHX). Therefore, the addition of CHX had minimal influences on puromycin decorporation such that puromycin decorporation were primarily attributable to changes in total MPB. A limitation also exists to using CHX in that it has been shown to inhibit MPB, albeit to a lesser extent than its effects on MPS [17]; therefore we suggest that use of CHX is neither required nor indicated, for this method.

The validity of this method was determined using treatments known to modulate MPB i.e. where a relative decrease in puromycin would indicate activation of MPB, and vice-versa. Serum starvation has been reported to activate MPB in muscle, primarily through activation of autophagy [18], while Dex and TNF-α have also been linked with activation of MPB in C2C12 cells [19], [20]. Our results showed that 24 h treatments of either serum starvation, Dex or TNF-α each caused a greater loss in puromycin labelling than untreated cells; similar changes were observed when CHX was included, indicating the effects were solely due to changes in MPB. We also tested the method with an inhibitor of protein breakdown, i.e. the proteasome inhibitor MG-132, and observed that there was a relatively lower loss of puromycin labelling with MG-132, consistent with an inhibition of MPB. These findings provide further substantiation that our method could accurately detect biologically driven changes in MPB in response to known catabolic factors.

Reported protein markers of MPB were measured to further verify the effects of each treatment. Both Dex and TNF-α caused upregulation of MAFbx and MuRF1 protein, consistent with previously reported effects of these compounds on activation of UPP-mediated protein breakdown [21], [22]. Unexpectedly, MG-132 also resulted in upregulation of MAFbx and MuRF1, however the finding that ubiquitinated protein levels were increased by MG-132 confirmed that proteasome-mediated MPB was inhibited. The observed decrease in MAFbx with serum starvation was also unexpected, although activation of autophagy with serum starvation may underlie the observed increases in MPB [18]. This was somewhat supported by the upregulation of ULK1 Ser555 phosphorylation at 4 h with serum starvation, which has previously been linked with activation of autophagy [23], [24] under conditions of stress. The three other conditions evaluated (Dex, TNF-α and MG-132) also showed increased ULK1 phosphorylation, with raptor phosphorylation, which is important for autophagy regulation via mTOR [25], also showing an increase with TNF-α and MG-132 treatment, indicating that these conditions may have also modulated autophagy pathways. Thus, measurement of known markers of MPB largely confirmed that our treatments were effectively influencing MPB. A clear advantage with the present method was that the same sample can be used to measure additional Western blot targets, including markers of MPB. Moreover, these findings on “static MPB markers” highlight the issues surrounding the use of MPB markers as a proxy for actual changes in MPB i.e. we observed MAFbx upregulation with MG-132, a treatment where proteasome-mediated degradation was inhibited, thus caution should be taken when using protein markers to assess MPB; similar to what has been shown with mRNA translation pathways poorly correlating to MPS [26], [27]. In addition, these results also emphasize the importance of measuring such static protein markers at multiple time points, since MAFbx protein had returned to control levels by 24 h with Dex, TNF-α and MG-132.

In summary, we present a novel proof-of-concept ‘puromycin decorporation’ method for assessing MPB via immunoblotting, validated using muscle cells *in vitro* – as an extension of the puromyocin incorporation method. This technique has potential for use in other cell types as well as *in vivo* studies, although new studies may require further optimisation in terms of puromycin doses and incubation periods. A major advantage of this method is that validation is relatively easily achievable and it does not require expensive or specialized techniques or equipment. We anticipate that this technique will allow for further insight into the role of MPB in skeletal muscle protein metabolism with health and conditions of muscle atrophy.
